# Three-dimensional modeling of removal torque and fracture progression around implants

**DOI:** 10.1007/s10856-018-6108-7

**Published:** 2018-06-30

**Authors:** Kohei Murase, Patrik Stenlund, Peter Thomsen, Jukka Lausmaa, Anders Palmquist

**Affiliations:** 1 Department of Mechanical Science and Engineering, Graduate School of Engineering, Nagoya University, Nagoya, Japan; 2 BIOMATCELL VINN Excellence Center of Biomaterials and Cell Therapy, Gothenburg, Sweden; 3 Department of Biomaterials, Institute of Clinical Sciences, Sahlgrenska Academy, University of Gothenburg, Gothenburg, Sweden; 4 RISE Research Institutes of Sweden AB, Gothenburg, Sweden

## Abstract

In the present study, a model for simulations of removal torque experiments was developed using finite element method. The interfacial retention and fracturing of the surrounding material caused by the surface features during torque was analyzed. It was hypothesized that the progression of removal torque and the phases identified in the torque response plot represents sequential fractures at the interface. The 3-dimensional finite element model fairly accurately predicts the torque required to break the fixation of acid-etched implants, and also provides insight to how sequential fractures progress downwards along the implant side.

## Introduction

Bone-anchored load-bearing implants are often used in surgery to restore a loss of function caused by disease or trauma. Osseous healing and the establishment of a direct contact (i.e., osseointegration) between the bone and implant is a prerequisite for clinical success both in oral [1], maxillofacial [2, 3], and orthopedic [4] applications. High long-term ( > 20 years) success rates have been shown for treatments of total or partially edentulous jaws using machined, screw-shaped titanium implants [5, 6**]**. In the last decades, large efforts have been directed to modifications of the surface physicochemical properties in order to improve the healing around implants [7]. Experimental in vivo studies have shown promising results for roughened implants, demonstrating increased bone growth and modified interfacial gene expression denoting cell recruitment, adhesion, differentiation, and bone remodeling [8–10].

In the development of novel implant materials and surface modifications, removal torque measurement is one of the most employed techniques to assess the degree of implant anchorage in bone. Such measurements have been used for studying fixation over time [11] as well as for comparisons between different materials [12] and implant surfaces [13, 14]. Implant surface roughness has been shown to be a factor that can affect both bone modeling and remodeling, resulting in a higher amount of bone at the implant surface and an increase of implant stability [15**–**19]. In addition, the hardness and stiffness of the surrounding bone tissue could increase due to surface treatment [20]. The geometrical features of the surface roughness in combination with tissue ingrowth also result in mechanical interlocking, which increases the removal torque [21, 22]. Further, different fracture scenarios have been described for rougher surfaces as compared to smoother surfaces [13, 23]. In order to separate the mechanical and biological effects of the surface roughness on the removal torque, a bench test was developed to eliminate the biological variances [21].

Finite element method (FEM) is a useful tool for analysis of the mechanics of the geometrically complex bone-implant system [24, 25]. For example, FEM has been used to illustrate and model how the implant surface [26] and the implant design and bone quality influence the stress/strain distribution in bone around implants [27] as well as how local tissue strains as a result of implant surface geometry affect the rate of osseointegration [28].

The objective of the current study was to develop a combined micro-macro finite model to simulate removal torque of cylindrical implants. The 3-dimensional finite element model was built to represent a previous experimental study, in which the effect of specific factors on the maximum removal torque of experimental titanium cylinders embedded in thermosetting polymers was evaluated [21]. Specific aims were to simulate the removal torque, how it is influenced by the geometry of the interface and to gain insights into the fracture progression around the implant during torque.

## Materials and methods

### Model geometries

**Fig. 1:**
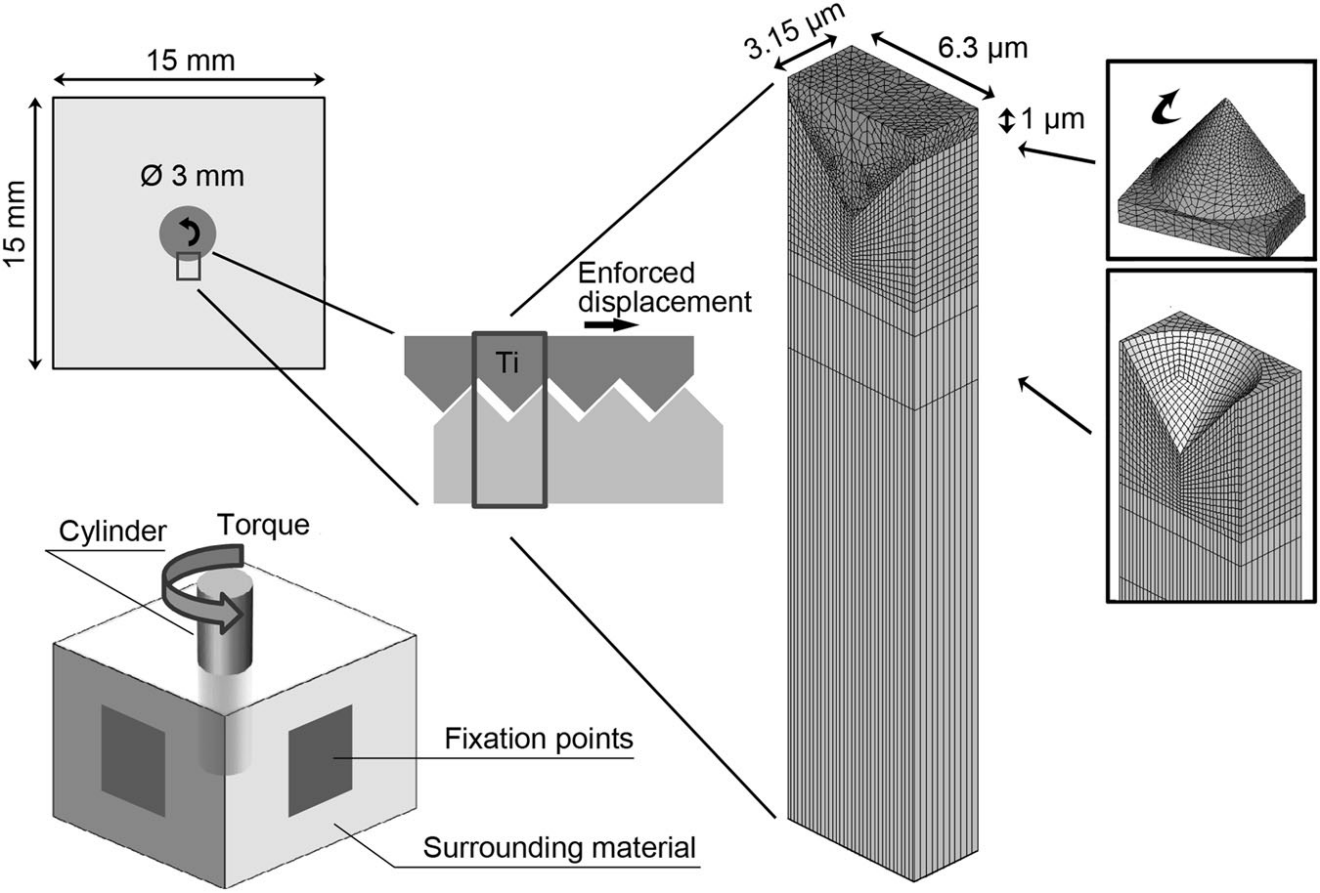
A schematic overview of the macro- and micro model geometries with the titanium cylinder and the surrounding material in dark and light gray, respectively

The model geometries and sizes are schematically illustrated in Fig. 1. A 3-dimensional macroscopic model was designed illustrating the full sized cylinder, 3 mm in diameter, embedded 9 mm in a block of homogeneous polymer of dimension 15 × 15 × 10 mm (*w* × *l* × *h*) used in a previous experimental study [21]. The block was divided in 20 layers, each 0.45 mm in height in the axial direction of the cylinder. Additionally, a 3-dimensional microscopic finite element model was designed with a conical shaped feature as a model of the acid-etched titanium. A gap ranging from 10–150 nm in size was introduced at the interface between the feature and the polymer as a way to simulate different contact situations. The size of the model was 3.15 × 6.3 × 6001 µm (*w* × *l* × *h*) in total. The topography of the microscopic model was assumed uniform and based on the surface roughness parameters characterized for the acid-etched surface in a previous experimental study [21]. The selected parameters were Sa: arithmetic mean deviation of the surface (849 nm), Sdr: developed surface area ratio (40%) and Sdq: root-mean-square slope of the surface (50 degree).

### Mesh

The microscopic model design was automatically meshed using hexahedral shaped 1st order elements aiming for uniform sizing of elements irrespective of the design. The mesh was refined until the volume of the finite element model and the corresponding computer aided design model showed good agreement. The microscopic acid-etched model had a total of 16,580 elements with 19,610 nodal points.

### Material properties

All materials were assumed homogenous isotropic and modeled as linear elastic solids. The elastic modulus of the implant was taken from titanium grade IV and was 113 GPa. The surrounding material was modeled with an elastic modulus of 3.76 GPa, taken from the polymer used in the bench study [21]. A Poisson’s ratio of 0.32 and 0.34 was assumed for the titanium and the surrounding material [29], respectively.

### Contact situations and boundary conditions

In the macroscopic model, not simulated by FEM, the bottom area of the implant cylinder was considered fixed when predicting the interfacial response when the implant was subjected to rotation, during this calculation the surrounding material was excluded and thus no boundary conditions necessary. However, the conditions of the previous experimental study it represents [21] underlies the conditions of the microscopic FE-model. In this experimental set-up the upper and lower sides were unconstrained while the center area (5 × 5 mm) on each of the four sides, perpendicular to the long axis of the cylinder, was modeled as fixed (Fig. 1).

The surrounding material in the microscopic model had the bottom surface fixed in all directions, the sides in the enforced direction modeled as continuous with tying constraints, and the sides perpendicular to the enforced direction fixed only in the normal direction. Frictionless contact was assumed at the interface and fractures in the surrounding material occurred when the von Mises equivalent stresses in the elements reached the yield strength of the polymer, 40 MPa, and modeled as removal of these specific elements.

### Analysis

The software package LS-DYNA V.970 (Livermore Software Technology Corporation, USA) was used to model nonlinear contact deformation in the microscopic model. The implant surface feature was displaced parallel with the interface and the reaction force in the displacement direction was calculated. The contact situation at the interface was altered due to high stress resulting in removal of elements. The process was iterated six times in order to determine the effect of a displacement corresponding to the experimental rotation of the cylinder [21]. The macro and micro models were then combined (Fig. 4) by layer-wise summation of the sum reaction forces determined by the microscopic simulations with regard to displacement, taking into account the difference in layer displacement identified in the macroscopic simulations due to implant material elasticity. The reaction force at different heights within each layer was approximated by the value at mean height and thus by mean displacement difference. When calculating sum reaction forces the reaction force at a given displacement was upscaled in terms of area with regard to each layer. Finally, the sum reaction force was converted to removal torque based purely on geometry and scaling after which comparison with the previous reported experimental results [21] was made.

### Validation of the models

The height of the surrounding material was evaluated by convergence analysis. The FEA result was validated against the result predicted in repeated simulations using alternative software (Abaqus 6.9, Dassault Systèmes, France). A layer-wise approach was used to compare the equivalent stresses in specific elements at the interface in both analyses.

## Results

### Finite element analysis

**Fig. 2:**
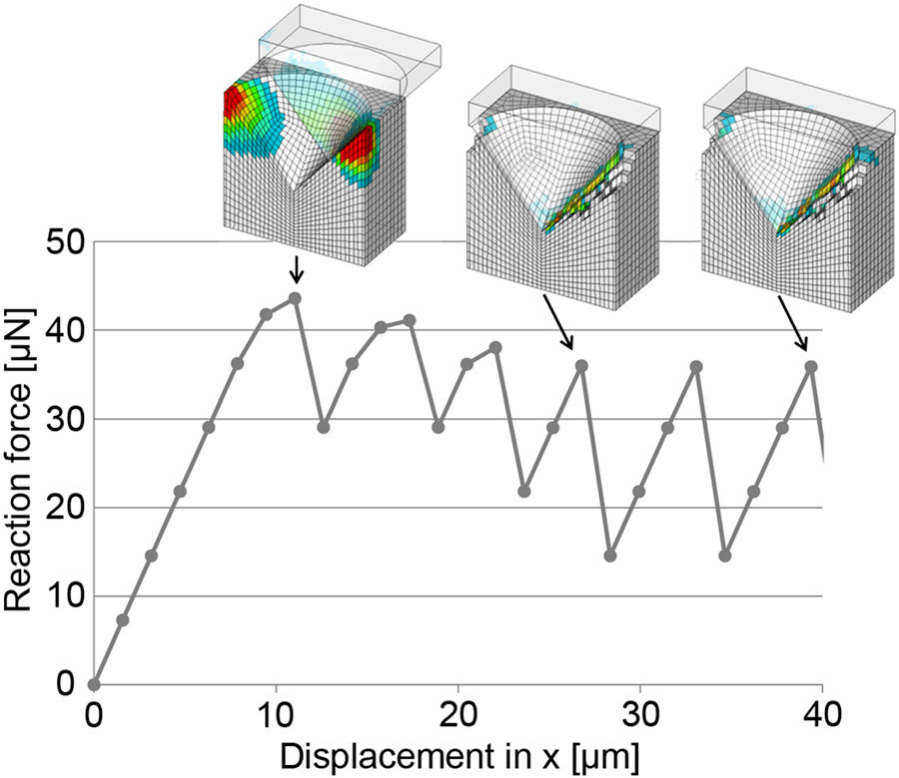
The reaction force in one segment using a 10 nm gap size for six subsequent iterations, presented with cross-sectional images for the 1st, 4th, and 6th iteration showing the stress distribution at the interface with fractured elements removed

**Fig. 3:**
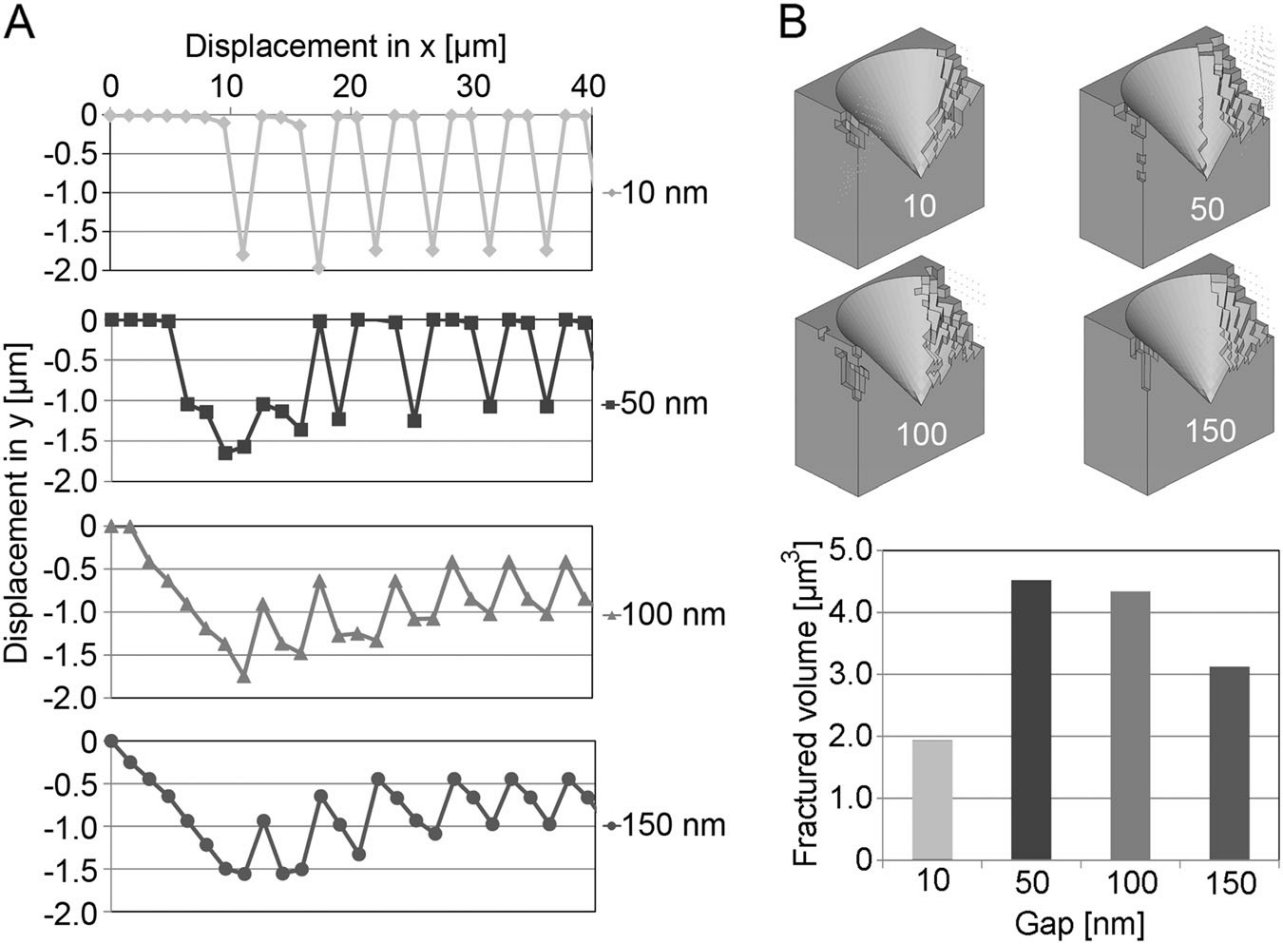
**(a)** Displacement of the elements perpendicular to the implant long axis (z) for different gap sizes (10–150 nm) when subjected to an enforced displacement along the *x*-axis, simulating a rotation. The simulation was terminated when no further fractures occurred. **b** The fractured interface for gap sizes 10–150 nm and the total volume loss after six iterations

**Fig. 4:**
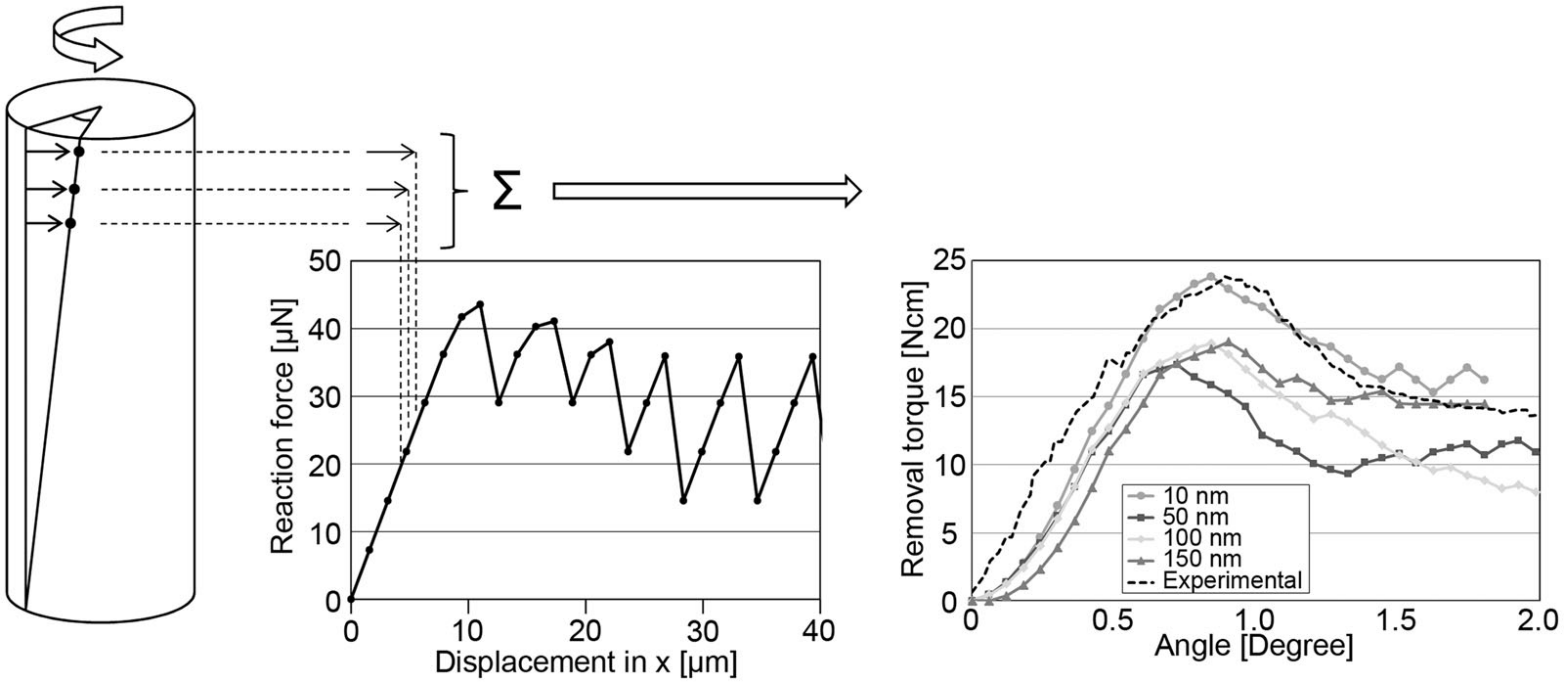
A schematic of the macro-model showing a change in displacement, indicated by arrows, at different heights along the implant caused by the enforced rotation. The linear difference in displacement defines the reaction force in each layer. The removal torque plot was derived by summation of the reaction forces in all layers. Additionally, the removal torque plot shows curves of the finite element analysis simulating different gap sizes in the interface region presented with one curve of the experimental bench study [21]

The macroscopic model simulated the interfacial response when the implant was subjected to rotation. Differences in displacement between the model layers were observed, decreasing linearly by 0.13 µm per layer, starting from the top, where the torque was applied, to the bottom. The total difference in displacement was 2.62 µm at a rotation corresponding to 0.1°. The microscopic model simulated the retention and fracture progression at the interface between the titanium surface and the surrounding material. The reaction force at the interface of the conical surface feature decreased with increasing number of iterations (Fig. 2). Introducing a gap at the interface between implant and surrounding medium in the microscopic model altered the contact area, which in turn affected the deformation (Fig. 3a) and fracture progression of the surrounding material (Fig. 3b). Conversion of the sum reaction force at the interface to removal torque showed torque levels similar to the experimental results (Fig. 4). Introducing a gap at the interface altered the fracture progression due to unique stress distribution in the surrounding material resulting in changed removal torque magnitudes (Fig. 4).

**Fig. 5:**
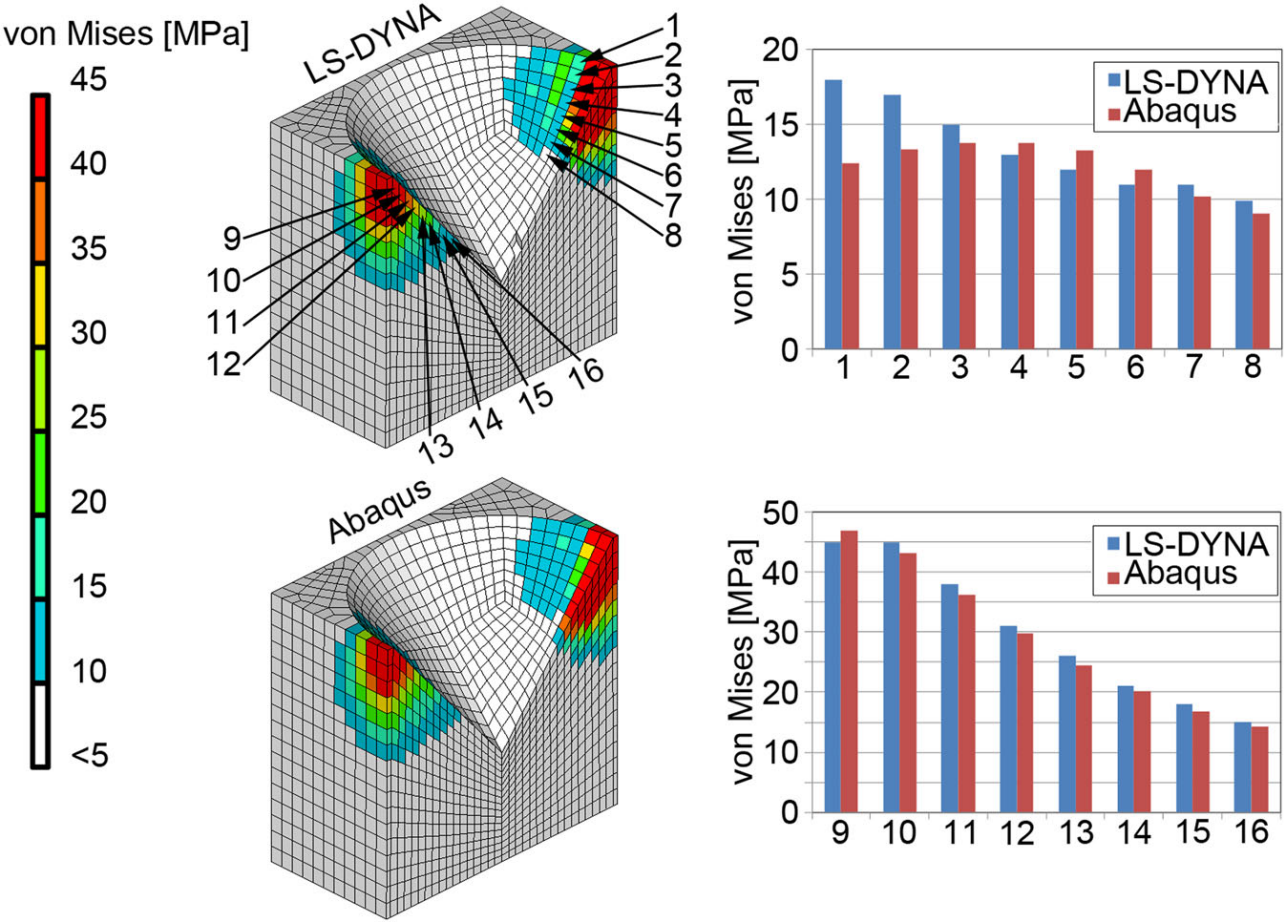
Validation of the FEA result by comparison of the equivalent stress in single elements at the interface determined by LS-DYNA and Abaqus

The validation of the FEA results showed equal levels of equivalent stress for the elements at the microscopic interface regardless of the software (Fig. 5). The convergence analysis confirmed an adequate model size, unaffected by the fixed boundary in the surrounding material.

## Discussion

The results of the present study showed the feasibility in building a 3-dimensional combined micro- and macro-model for predicting the removal torque and the fracture scenario occurring around acid-etched implants with a cylindrical geometry. Removal torque experiments are often used to evaluate implant stability in preclinical in vivo models, where increased removal torque values are typically observed around rough implants as compared to smooth implants [30]. Different fracture scenarios have also been discussed based on the shapes of the load-deformation plots obtained during measurements [14, 19], where smoother implants typically have a separation occurring at the immediate interface, whereas rougher implants have fractures in the bone tissue. The conical surface feature used to model the acid-etched surface can be considered a gross approximation but showed fractures in the embedding medium progressing along the implant length starting at the top, which is in agreement with visual observation during a previous bench study [21]. Furthermore, a similar load-deformation plot in conjunction with visible fractures in the bone tissue as judged by histology has been described for acid-etched implants after removal torque test in vivo [31].

Previous mathematical models proposed to predict interfacial shear strength have been done in two dimensions and later extended to three dimensions, with a local [32] and global [22] model. The models were based on the surface roughness of the implants with the mean slope of the 2D profile identified as a main parameter for the retention, but also the features height (*R*_a_) and mechanical properties of the surrounding medium were considered [22]. Clinical dental implants are screw shaped, and depending on whether they are modified by anodic oxidation, laser ablation, sandblasting, or acid etching they have been shown to have different shapes of the surface features [33] but quite similar roughness in terms of Sa [7]. It is difficult to fully describe a complex surface topography mathematically, even though 59 different surface roughness parameters have been described [34]. Further, accurate measurement of the surface roughness of complex shaped threaded implants is associated with limitations of different techniques [35] as well as the need for filtering to remove macro shape and waviness [36].

The simplifications made in the model used here needs to be pointed out. The topography of the acid-etched surface was modeled as homogenous single size conical protruding features matching the selected etched surface roughness parameters. Others have described models for surface pits [37], however it is believed from the literature on implant surfaces [33], that the true surface contains both types of surface features. The size of the feature and the layer height was not an even multiple and not considered when the models were combined since the area of the features it concerns (layer interface region) corresponds to <1% of the total area. However, the area of those features was still included when calculating sum reaction forces in specific layers. Also, the reaction force at different heights within each layer was approximated by the value at mean height and mean displacement difference. Another simplification of the current model was that a non-adhesive contact was assumed limiting the model to pure mechanical interlocking by surface geometrical features that contribute to reaction forces and possibly also fractures. This limits the model to implant surfaces showing a fracture-like break-point in removal torque, and not suited for smoother surfaces where most likely the friction is the dominating factor [14]. However, as the model was built on the reaction forces in the micro model and summed with a linear delay equal to the difference in layer displacement makes the combined model variable. This allows for unrestricted freedom to modify the shape of the surface features, combining features of different shapes and sizes as well as changing the mechanical properties of the surrounding medium. Changing the implant geometry to for example a screw-shape would result in different reaction force responses at the surface due to changed surface orientation. The observed differences between experimental and simulated removal torque are likely to be explained by the simplifications described above.

Further, osseointegrated implants typically display nonintegrated regions were there exist a gap between bone and implant. By introducing a gap at the interface the model simulates a more realistic situation of an osseointegrated state. Moreover, it brings understanding to the effect of non-contact situations on removal torque, even if the model can be considered a gross simplification. The gap was shown to alter the contact situation, which introduced changes in the fracture progression at the interface resulting in successive decreased reaction forces and thereby lower removal torque value. The effect of a gap at the implant interface has previously been discussed [22] and was concluded to lower the interfacial shear strength considerably as a result of the lower contact area. The previously observed variation in removal torque in an experimental bench study [21] may have been caused by differences in the contact situation for the complex surface topography. The removal torque magnitude measured in bone will reflect mechanical properties of the bone and depend on the interfacial conditions with regard to the heterogeneous topography as well as the implantation site, and the direction and speed loads are applied. In the typical preclinical in vivo models the implants are often evaluated using tibia or femur sites, and when torque is applied the loads will be directed parallel with the implant surface and thus both longitudinally and transversally in the bone. Therefore, a more complex fracture pattern and reaction force progression can be expected. For small loads the bone is likely to display linear deformation trends but since bone fractures do occur the bone´s viscoelastic properties will to some extent affect the results. Before the model can be used for in vivo simulations it needs to be validated and possibly revised to accurately model bone anisotropy and viscoelastic properties, updated with more complex implant surface geometries, simulating additional, clinically relevant situations. Micro-CT bone data from preclinical experimental studies can provide information on the amount and mineral density of the bone to use in the macro-model. Such approach would create a more realistic model enabling direct comparison with the removal torque data generated in vivo.

Identifying how the complex interfacial fractures occur is important in order to understand how to design and improve implant surfaces to reduce stress concentrations, fractures and improve implant stability.

## Conclusions

A 3-dimensional finite element model was developed, which could fairly accurately reproduce the removal torque required to break the acid-etched implant fixation. The model provides a plausible explanation to how the fracture progression at the interface occurs, involving sequential fractures with loosening from the top of the implant to the bottom.
